# Communicating hydrocephalus associated to ventral leptomeningeal invasion leads to precocious death in a glioblastoma orthotopic xenograft model

**DOI:** 10.1093/noajnl/vdaa099

**Published:** 2020-08-17

**Authors:** Arnaud Lombard, Marina Digregorio, Natacha Coppieters, Emmanuel Di Valentin, Alexandre Hego, Didier Martin, Bernard Rogister

**Affiliations:** 1 Laboratory of Developmental Neurobiology, GIGA-Neuroscience, University of Liège, Liège, Belgium; 2 Department of Neurosurgery, CHU and University of Liège, Liège, Belgium; 3 GIGA Viral Vectors Platform, University of Liège, Liège, Belgium; 4 GIGA Cell Imaging Platform, University of Liège, Liège, Belgium; 5 Department of Neurology, CHU and University of Liège, Liège, Belgium

**Keywords:** glioblastoma, hydrocephalus, in vivo model, leptomeningeal invasion

**Key Points**

- Skull base infiltration by GBM cells in an orthotopic xenograft mouse model of GBM.
- Subsequent hydrocephalus and precocious death.

*In vivo* models are essential for the study of glioblastoma (GBM). Patient-Derived Orthotopic Xenograft (PDOX) using GBM cells in immunodeficient mice classically results into 2 main scenarios of tumor development, either intracerebral or intraventricular. In the present brief report, we have used brain clarification and lightsheet imaging to highlight the occurrence of a communicating hydrocephalus associated to the ventral leptomeningeal invasion by cancer cells, leading to a decreased survival. We believe that this uncommon event should be taken into account for interpretation of forthcoming survival manipulations in preclinical studies.

Glioblastoma (GBM) is the most frequent malignant primary brain tumor in adults, well-known for its poor prognosis despite multimodal therapies.^1^ In this context, multiple novel therapeutic strategies are tested in preclinical studies, in order to increase survival in vivo. Currently available mouse models include human GBM cell line orthotopic xenografts, Patient-Derived Orthotopic Xenograft (PDOX), Genetically Engineered Mouse Models (GEM) and syngeneic mouse models.^2^ For PDOX, tumor-associated immune response is impeded due to the use of immunodeficient mice, but the tumor development seems to recapitulate more accurately the genetic and phenotypic features of the disease in comparison to the other mouse models.^2^ In xenograft models, Irtenkauf et al. reported the possible development of a tumor either in the brain parenchyma or in the ventricles, depending on the site of injection.^3^ Using brain clarification and lightsheet imaging after PDOX, we report a third possible scenario.

The human primary GBM culture GB1 was established from a resected newly-diagnosed tumor (WHO grade IV, *IDH* wild-type, *p53* wild-type, MGMT non-methylated) obtained after informed patient and cultivated as neurospheres.^4^ GB1 cells were transduced to express RFP before being grafted orthotopically in nude mice.^4^ Clarification of each brain hemisphere was performed separately, as described by Tomer et al.^5^ Images of each hemisphere were obtained using a dual illumination lightsheet fluorescence microscope (Zeiss®). Images were stitched, reconstructed, and analyzed using Imaris® software (version 9.5.0). This study has been approved by the ethical committee of the University of Liège (IRB 2197).

**Figure 1.**
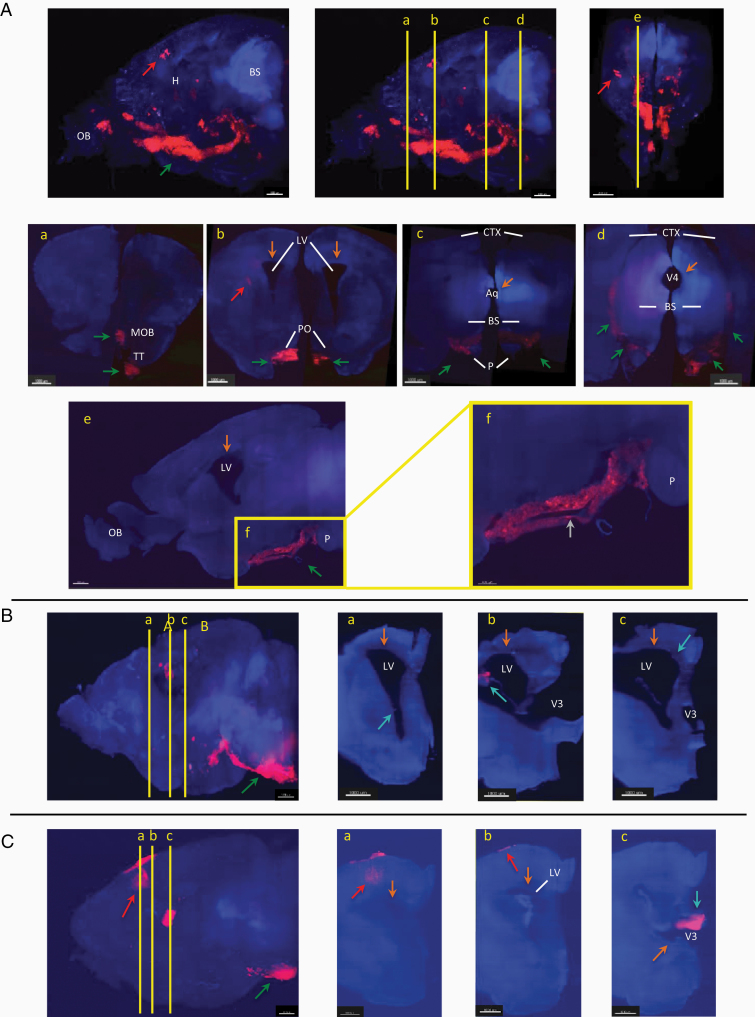
Pattern of dissemination of glioblastoma (GBM) tumor cells associated with communicating hydrocephalus after PDOX in 3 specimens. Red arrows show GBM cells engrafted in the striatum. Green arrows show ventral tumoral invasion. (A) Profile (left-middle) and top (right) views of the brain of specimen I, after clarification. The red signal consists of RFP-positive tumor cells. a. Coronal section at the olfactory bulb (OB): At the most anterior part of the brain, tumor cells are localized at the ventral part (green arrows) of the main olfactory bulb (MOB) and the tenia tecta (TT). b. Coronal section at the site of injection: Few GBM cells are found in the striatum (site of injection, red arrows), while numerous tumor cells (green arrows) are adjacent to the ventral walls of the medial pallidum, of the optic tract and of some ventral hypothalamic nuclei, particularly the preoptic nuclei (PO). c–d. Coronal sections through the brainstem (BS) at the level of the aqueduct (Aq, section c) and the fourth ventricle (V4, section d): GBM cells invaded the subarachnoid spaces between the pons (P) and the midbrain medially (section c, green arrows), but also between the midbrain and the cortex (CTX) laterally (section d, green arrows). e–f. Sagittal sections through the right lateral ventricle (LV): GBM cells localized in the ventral leptomeningeal spaces (green arrow) do not present invasiveness regarding adjacent parenchyma and surround ventral cerebral vessel (gray arrow). (B) Profile view of the brain of Specimen II (left) and coronal sections of its right hemisphere (middle and right), after clarification. a–c. Sections around the site of injection. Some tumor cells invaded the wall of the LV and the choroid plexus (blue arrows). (C) Profile view of the brain of Specimen III (left) and coronal sections of its right hemisphere (middle and right), after clarification. a–c. Sections around the site of injection. Some tumor cells invaded the choroid plexus of the third ventricle (blue arrow). Abbreviations: hemispheres (H), third ventricle (V3), pons (P), preoptic nucleus (PO). Arrows: Red, site of injection; Orange, dilation of the ventricular system; Green, ventral tumor invasion; Blue, subventricular/ventricular invasion; gray, ventral cerebral vessel.

In a first study (N1), a single mouse (specimen I) presented severe signs of suffering (curved back and weight loss of more than 15% in 3 days) 1 month after being grafted, while the remaining 9 mice developed similar symptoms only 3 months after the engraftment. This symptomatic mouse was hence precociously sacrificed. After brain clarification (Figure 1A), images of the prematurely perfused mouse brain were taken with a lightsheet microscope and revealed the presence of few tumor cells in the right striatum and cortex near the site of injection (Figure 1A, section b, red arrows). Unexpectedly, we observed a severe dilation of the whole ventricular system without obstruction by tumor engraftment in the ventricles, which defines a communicating hydrocephalus (Figure 1A, section b–d, orange arrows). Moreover, we highlighted an important tumor infiltration in the ventral part of the brain and the brainstem (BS) (Figure 1A, green arrows).

The tumor cells observed at the ventral part of the forebrain are forming a symmetrical pseudo-mass of 300–500 μm thickness, adjacent to the ventral part of not-invaded brain and forebrain, which extend anteriorly along the ventral walls of the optic tract and the olfactive bulb (Figure 1A, section a–b), and posteriorly between the midbrain and the pons medially (Figure 1A, section c), between the midbrain and the cortex laterally (Figure 1A, section d). In this posterior progression, it is visible that tumor cells are fulfilling the subarachnoid spaces, surrounding ventral cerebral vessels, and do not present invasive behavior regarding the adjacent brain and forebrain (Figure 1A, section e–f). Hence, the tumoral spreading corresponds to leptomeningeal dissemination.

In a second study (N2) using the same GBM cells, we highlighted the occurrence of tumoral leptomeningeal dissemination in 2 additional specimens. The second specimen (II) presented identical suffering symptoms and we observed the same pattern of ventral dissemination with subsequent severe hydrocephalus and precocious death at 6 weeks, although tumor cells engraftment in the striatum was not detected (Figure 1B). The third specimen (III) was perfused precociously at 8 weeks because of significant weight loss (>15% in 3 days). Images revealed the engraftment of a limited amount of tumor cells in the cortex surrounding the site of injection and in the wall of the third ventricle (blue arrow) and the invasion of the ventral part of the brain/brainstem with moderate dilation of the ventricular system (Figure 1C). This corroborates the fact that specimen III presented fewer symptoms than specimens I and II.

The mechanism of ventral leptomeningeal infiltration in PDOX remains uncertain. Two hypotheses could be considered and might not be exclusive. (1) Due to reflux during cell injection, tumor cells might not graft in the brain and might drift in the declivitous ventral part of the brain. In this condition, the presented observation would rather result from a technical issue at the engraftment and would therefore not be expected in GEM GBM models. (2) It is possible that tumor cells follow the cerebro-spinal fluid flow to the ventral skull base as recently reported by Ahn et al.^6^ The accumulation of tumor cells in the ventral subarachnoid spaces could, therefore, explain the development of communicating hydrocephalus. In GBM patients, the onset of communicating hydrocephalus is a late event, usually reported after surgery and irradiation, occurring in less than 10% of patients.^7^ Its association with leptomeningeal dissemination remains controversial.^7,8^

In conclusion, communicant hydrocephalus associated to ventral leptomeningeal invasion leading to precocious death after PDOX is a rare event, as shown by the 3 out of 35 mice (8,6%) affected in the present report. Irtenkauf et al. reported the occurrence of intraventricular tumor in around 10% of mice, using the same coordinates as us.^3^ Ventral leptomeningeal infiltration and intraventricular tumors both seem to be associated with shorter survival. In case of early onset of suffering symptoms, we would recommend MRI realization, looking either for the development of communicating hydrocephalus associated to the restricted development of a tumor mass in the striatum or for the formation of an intraventricular tumor mass. Taken together, those findings suggest that preclinical survival studies using PDOX should confirm the localization of the tumor in order to adequately compare mice survival and to determine more accurately if tested therapies display significant improvement.
